# Synergistic rhizobacteria enhance physio-biochemical resilience and sustain tomato yield under drought stress

**DOI:** 10.1038/s41598-026-51973-2

**Published:** 2026-05-13

**Authors:** Preeti, Pradeep Kumar Rai, Danish Mushtaq Khanday, Sadiya M. Choudhary, Monika Singh, Sadaf Wani, Pradeep Kumar, Nazim S. Gruda, Gyanendra Kumar Rai

**Affiliations:** 1 Division of Soil Science and Agricultural Chemistry, Sher-e-Kashmir University of Agricultural Sciences and Technology of Jammu, Chatha, 180009 JK, UT India; 2 Division of Plant Breeding and Genetics, Sher-e-Kashmir University of Agricultural Sciences and Technology of Jammu, Chatha, 180009 JK, UT India; 3 Institute of Biotechnology, Sher-e-Kashmir University of Agricultural Sciences and technology of Jammu, Chatha, 180009 JK, UT India; 4 G.L. Bajaj Institute of Technology and Management, Greater Noida, 201306 Utter Pradesh India; 5 Division of Integrated Farming System, ICAR-Central Arid Zone Research Institute, Jodhpur, 342003 India; 6 University of Bonn, Bonn, Germany

**Keywords:** Abiotic stress response, Root-microbe interaction, Cellular protection, Stress mitigation, Microbiology, Plant sciences

## Abstract

Beneficial rhizobacteria can enhance plant growth and stress resilience through multiple, complementary mechanisms. In this study, we investigated the combined effects of *Azotobacter chroococcum*, *Pseudomonas putida*, and *Bacillus subtilis* on tomato plants subjected to drought stress. The primary objective was to assess whether a combined bacterial inoculation could mitigate the negative impacts of drought on tomato growth and productivity. We hypothesized that the consortium would act synergistically to improve drought tolerance by enhancing physiological performance and biochemical defense systems, including photosynthetic activity and antioxidant enzyme responses. The combined application of beneficial rhizobacteria significantly increased total chlorophyll content from 0.85 to 1.70 mg g⁻¹ FW and relative water content from 55.41 to 72.06%, while maintaining higher photosynthetic pigment levels than drought-stressed controls. Biochemical analyses revealed markedly higher activities of antioxidant enzymes, with superoxide dismutase increasing to 35.4 µmol min⁻¹ mg⁻¹ FW, catalase to 74.6 µmol min⁻¹ mg⁻¹ FW, and peroxidase to 0.89 µmol g⁻¹ FW, indicating more effective mitigation of drought-induced oxidative stress. All individual rhizobacterial treatments significantly increased tomato yield relative to drought stress, reaching 0.81, 0.86, and 0.88 kg plant⁻¹ under inoculation with *(A) chroococcum*, *P. putida*, and *(B) subtilis*, respectively. The bacterial consortium produced the highest yield of 0.94 kg plant⁻¹. Overall, these findings demonstrate that synergistic plant–microbe interactions can substantially enhance drought tolerance and productivity in tomato. Future studies should examine the long-term stability and field performance of microbial consortia, their interactions with native soil microbiomes, and their scalability for sustainable crop production in water-limited agroecosystems.

## Introduction

Tomato (*Solanum lycopersicum* L.) is one of the most widely cultivated vegetables globally, prized for its exceptional nutritional and economic value. Its fruits are rich in lycopene, a powerful antioxidant with health benefits, including anticancer properties. Beyond its nutritional appeal, the tomato’s vibrant color and culinary versatility have made it a staple in diets worldwide. However, the productivity of this beloved crop is often hindered by abiotic stresses, with drought among the most significant challenges^1,2^.

Drought stress negatively impacts plant growth by disrupting physiological and biochemical processes, including photosynthesis, nutrient uptake, and water relations, ultimately reducing growth and yield^3,4^. Roots play a crucial role in acquiring the water and nutrients plants need to sustain their growth. Under drought stress, they often change their architecture and function due to water limitations^1^. This makes them particularly vulnerable to the adverse effects of drought, which hinders the plant’s ability to thrive in such conditions. In response to these challenges, microbial applications have emerged as a promising strategy to help tomato plants cope with the harmful effects of drought stress. Beneficial microbes influence key physiological and biochemical processes to enhance drought resilience. They support osmotic adjustment by accumulating compatible solutes such as proline, sugars, and betaines, which help maintain cell turgor and reduce wilting^5^. Additionally, microbes bolster the plant’s antioxidant defense system by increasing the activity of enzymes such as superoxide dismutase, catalase, and peroxidase, thereby scavenging reactive oxygen species and mitigating oxidative damage^6,7^.

Microbial communities also play a crucial role in hormonal regulation; some produce 1-aminocyclopropane-1-carboxylate deaminase, an enzyme that lowers ethylene levels, alleviating stress-induced growth inhibition^8^. Moreover, under drought conditions, nutrient acquisition, often hampered by water scarcity, is enhanced by microbes that solubilize phosphorus, fix nitrogen, or produce siderophores, thereby increasing nutrient availability to the plant^9^. Certain microbes can also modify root architecture, promoting root growth and development, thereby increasing root surface area and improving water and nutrient uptake under drought conditions^10^.

Among various agronomic strategies, inoculation with beneficial microbes represents a useful, sustainable approach. Soil microorganisms, such as bacteria, fungi, archaea, and Oomycetes, interact with plants to enhance their tolerance to water-deficit conditions^11–13^. Specifically, plant growth-promoting rhizobacteria (PGPR) can improve plant resilience to drought stress through their diverse functions. For instance, *Azotobacter chroococum* is known for its nitrogen-fixation capabilities, which will enhance nutrient availability for plants under stress^14,15^. *Pseudomonas putida* produces phytohormones such as indole-3-acetic acid, which promote root development and improve water uptake^16^. *Bacillus subtilis* can induce systemic resistance in plants, improving their overall resilience to stress^17^.

While individual plant growth–promoting rhizobacteria (PGPR) are widely recognized for their capacity to alleviate drought stress, comparatively little attention has been given to the potential benefits of their combined application. In particular, the interactive and synergistic effects of *Azotobacter chroococcum*, *Pseudomonas putida*, and *Bacillus subtilis* on drought-stressed tomato plants remain insufficiently explored. We hypothesize that the simultaneous application of these PGPRs will confer greater drought tolerance than single-strain inoculations by collectively enhancing morphological development, physiological performance, and biochemical stress-defense mechanisms.

Accordingly, this study aims to investigate the effects of a PGPR consortium on physiological performance and biochemical stress-defense mechanisms associated with drought tolerance under water-limited conditions. In addition, the study seeks to determine whether these consortium-induced responses translate into improved plant growth and yield compared with single-strain inoculations and non-inoculated controls. The results are intended to support the development of sustainable, microbe-based strategies for improving tomato production in water-limited agroecosystems.

## Materials and methods

### Experimental setup and plant materials

The experiment was conducted during winter season in the plastic greenhouse of the Advanced Centre for Horticulture Research (ACHR), Udheywala, Jammu, using sandy loam soil (0–20 cm layer) with a pH of 7.47, electrical conductivity of 0.19 dS/m, organic carbon of 0.53%, nitrogen 131.45 mg/kg, phosphorus 9.06 mg/kg, and zinc 0.31 mg/kg, which was autoclaved at 121 °C for two hours before being filled into 30 cm height and 40 cm diameter pots, each containing 15 kg of soil.

Tomato seedlings (*Solanum lycopersicum* L. var. ‘Pusa Ruby’) were surface sterilized with 0.1% sodium hypochlorite and rinsed with Milli-Q water. The bacterial cultures of *Azotobacter chroococcum*, *Pseudomonas putida*, and *Bacillus subtilis* were grown separately and adjusted to a uniform cell density of approximately 10⁸ CFU mL⁻¹. The concentration was standardized by measuring the optical density at 600 nm (OD₆₀₀ ≈ 0.8) with a UV–Vis spectrophotometer and was further confirmed by serial dilution and plate count methods. For individual treatments, seedlings were inoculated with the respective bacterial suspension. For the consortium treatment, equal volumes of each bacterial culture were mixed in a 1:1:1 ratio to prepare the combined inoculum. The seedlings were then inoculated by dipping their roots in the respective bacterial suspensions for 30 min prior to transplanting.

The experimental design included six treatments: control (normal conditions), drought stress, and drought stress with bacterial inoculants (*Azotobacter chroococum* + drought, *Pseudomonas sp.* + drought, *Bacillus subtilis* + drought, and a bacterial consortium + drought), all arranged in a completely randomized design with five replicates. Pots were kept in a growth chamber under a 16-hour day/8-hour night photoperiod, 300 µmol m^−^²s^− 1^ light intensity, 22 °C/18°C day/night temperatures, and 60–70% relative humidity. Soil moisture was maintained at 35 ± 5% under normal conditions and reduced to 8 ± 2% in drought using a gravimetric method. After 21 days of seedling establishment, plants were subjected to water deficit stress for 5 days before being carefully uprooted and transported to the laboratory for further analysis.

### Assessment of physiological parameters

#### Chlorophyll contents

Chlorophyll (a and b) and total chlorophyll content were estimated in fully expanded leaves using the method described by Ashraf and Iram^18^. Leaf samples (0.2 g) were incubated in 10 mL of an 80% aqueous acetone solution and then centrifuged at 14,000 g for 5 min. The absorbance of the extracts was measured at 653 nm and 645 nm. Chlorophyll a, b, and total chlorophyll content were determined using a UV spectrophotometer (Model UV, Unicam, Cambridge, UK). Chlorophyll concentrations were calculated using the following equations:

Chlorophyll a (mg g⁻¹ FW) = (12.7 × A₆₅₃ − 2.69 × A₆₄₅) × V/(1000 × W).

Chlorophyll b (mg g⁻¹ FW) = (22.9 × A₆₄₅ − 4.68 × A₆₅₃) × V/(1000 × W).

Total chlorophyll (mg g⁻¹ FW) = (20.2 × A₆₄₅ + 8.02 × A₆₅₃) × V/(1000 × W).

where A₆₅₃ and A₆₄₅ represent absorbance at 653 nm and 645 nm, respectively, V is the extract volume (mL), and W is the fresh weight of the sample (g).

#### Malondialdehyde (MDA) contents

MDA content in tomato leaves was estimated using the method described by Stewart and Bewley^19^. Briefly, a 0.2 g leaf sample was homogenized with 2 mL of ethanol and centrifuged at 10,000 g for 10 min. The enzyme extract (1 mL) was then mixed with 2 mL of a thiobarbituric acid (TBA, 0.5%) and trichloroacetic acid (TCA, 20%) mixture. The mixture was boiled for 30 min, cooled immediately, and centrifuged again at 10,000 rpm for 5 min. MDA content was determined by measuring the non-specific absorption difference at 600 nm and 532 nm.

#### Determination of antioxidant enzymes

Enzyme activities were measured by extracting fresh tomato leaves with 5 mL of phosphate buffer (50 mM, pH 7.8) containing 0.5 mM EDTA and 2% polyvinylpyrrolidone (PVP-40). The homogenate was centrifuged at 15,000 g for 15 min at 4 °C, and the supernatant was used for further analysis. Superoxide dismutase (SOD, EC 1.15.1.1) activity was determined using the method by Beyer and Fridovich^20^, based on the photochemical reduction of nitroblue tetrazolium. One enzyme unit was defined as the amount of enzyme that inhibits 50% of the photoreduction of nitroblue tetrazolium at 560 nm. Catalase (CAT, EC 1.11.1.6) activity was calculated using Hadwen^21^ method. Ascorbate peroxidase (APX, EC 1.11.1.9) activity was assessed by monitoring the decomposition of hydrogen peroxide (H₂O₂) at 290 nm for 2 min, with absorbance changes recorded as described by Hong et al.^22^.

#### Osmolyte (Proline content)

Proline concentration in leaves was measured using the ninhydrin method described by Raza and Khan^23^. Leaf samples (100 mg) were dissolved in 2 mL of 3% sulfosalicylic acid solution and centrifuged at 13,000 rpm for 10 min. One milliliter of the supernatant was mixed with 1 mL of freshly prepared ninhydrin solution and 1 mL of glacial acetic acid in a test tube. The mixture was heated at 100 °C in a water bath for one hour, then allowed to cool to room temperature. After cooling, 2 mL of toluene was added, and the mixture was vortexed for 20 s. The test tubes were left to stand for at least 10 min to allow the toluene and aqueous phases to separate. The absorbance of the toluene phase was measured at 520 nm using a spectrophotometer. Proline concentration was determined using a standard proline curve and expressed as µmol g^− 1^ fresh weight (fw).

### Assessment of growth characters

At harvest, tomato plants were gently removed from the pots and carefully rinsed with distilled water to remove adhering soil particles. Plant height (cm) was measured from the soil surface up to the apical meristem, while root length (cm) was recorded from the collar region to the tip of the longest root.

Fresh weights of shoots and roots (g) were recorded immediately after harvest using a digital analytical balance. The plant samples were subsequently dried in a hot-air oven at 70 °C until constant weight was attained, after which shoot and root dry weights (g) were determined.

The root-to-shoot ratio was calculated on a dry weight basis as the ratio of root dry weight to shoot dry weight. Relative water content (RWC) was estimated using fully expanded leaves following the method described by Barrs and Weatherley (1962). Leaf fresh weight was measured immediately after sampling, and the leaves were then immersed in a 5 mM CaCl₂ solution for 24 h to achieve full turgidity. The leaves were then oven-dried at 70 °C for 72 h to obtain dry weight. Relative water content was calculated using the formula described by Gowtham et al.^24^.

 RWC=(Fresh weight-Dry weight%Turgid weight-Dry weight)100

### Assessment of yield parameters

Yield and yield-related traits were recorded at physiological maturity using three biological replicates per treatment. The number of flowers per cluster was counted manually from tagged inflorescences, while the number of fruits per cluster was recorded at fruit maturity. The total number of fruits per plant was determined by summing fruits harvested from successive pickings throughout the cropping period.

Fruit set percentage was calculated as the proportion of flowers that successfully developed into fruits, following standard horticultural evaluation procedures^25,26^, using the following formula:

$$\:\mathrm{Fruit\:set\:(\%)}=\frac{\mathrm{Number\:of\:fruits\:per\:plant}}{\mathrm{Number\:of\:flowers\:per\:plant}}\times\:100$$

Yield per plant (kg plant⁻¹) was calculated by weighing all fruits harvested from each plant on a digital analytical balance and summing their weight. Yield measurements were expressed on a per-plant basis to enable accurate comparisons among treatments under controlled drought conditions, as commonly used in tomato drought-stress studies^27^.

All yield and yield-related observations, including the number of flowers per cluster, the number of fruits per cluster, the number of fruits per plant, the fruit set percentage, and the yield per plant, were recorded at harvest maturity to ensure consistency and minimize variation due to developmental stage differences among treatments.

### Statistical analysis

Statistical analysis of the data was performed using R Studio software (R Studio Team, 2021). Normality and homoscedasticity were evaluated using the Shapiro-Wilk test and Bartlett’s test, respectively, through the default functions in R Studio. For data that followed a normal distribution, a one-way ANOVA model was applied, considering five sources of variation: irrigation, water deficit, microbial inoculation, and media. Statistical significance was determined using the D’Agostino test of skewness on the residuals, followed by post-hoc Tukey’s Honest Significant Difference test.

## Results

### Physiological status, membrane integrity, and oxidative damage

**Fig. 1:**
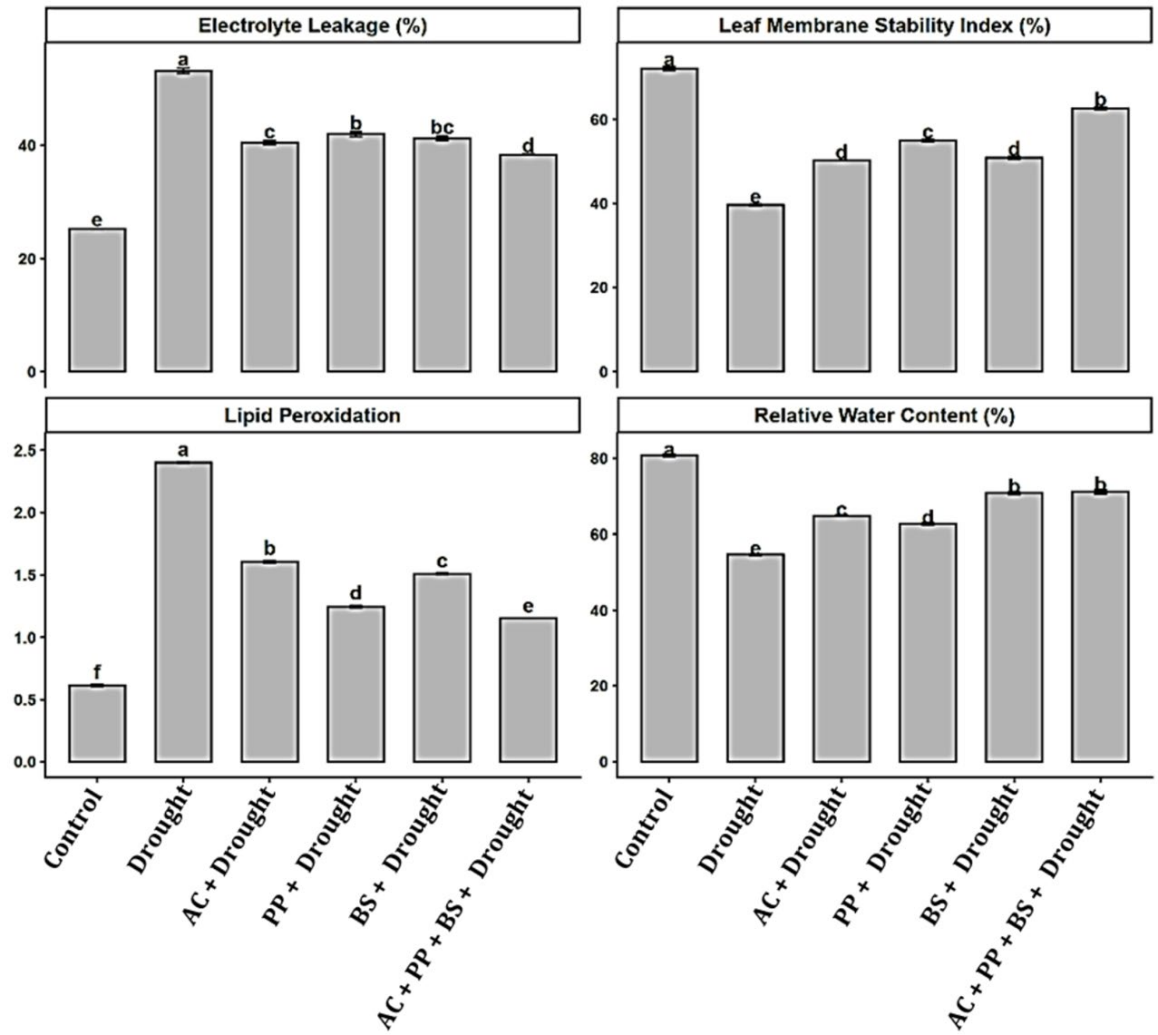
Effect of rhizobacterial application on (**A**) electrolyte leakage (**B**) leaf membrane stability index, (**C**) lipid peroxidation and (**D**) relative water content of tomato under drought stress. Bars represent mean values ± standard error (SE) of three biological replicates (*n* = 3). Different lowercase letters above the bars indicate significant differences among treatments according to the LSD test at *p* ≤ 0.05.

Drought stress induced severe physiological impairment in tomato plants (Fig. 1), as evidenced by significant reductions in relative water content (RWC) and membrane stability, along with marked increases in electrolyte leakage and lipid peroxidation (Fig. 1). Relative water content declined from 80.21% in the control to 55.41% under drought, corresponding to a 30.9% reduction. Inoculation with *Azotobacter chroococum* under drought increased RWC to 65.12%, representing a 17.5% improvement over drought conditions. Similarly, *Pseudomonas putida* and *Bacillus subtilis* raised RWC to 62.87% (13.5% increase) and 70.84% (27.8% increase), respectively, compared with drought-stressed plants. The combined consortium AC + PP+BS achieved the highest RWC (72.06%), a 30.1% increase over drought conditions, and it was only 10.2% lower than the well-watered control.

Electrolyte leakage increased sharply under drought conditions, rising from 25.12% in the control to 53.82%, representing a 114.3% increase. Application of *(A) chroococum*, *P. putida*, and *(B) subtilis* under drought stress reduced electrolyte leakage to 40.63%, 42.28%, and 41.21%, corresponding to reductions of 24.5%, 21.5%, and 23.4%, respectively, relative to drought. The consortium exhibited the lowest electrolyte leakage (36.11%), representing a 32.9% reduction compared with drought and a further 10.5–14.6% decrease compared with individual inoculations.

Leaf membrane stability index (LMSI), which reflects the stability and integrity of cellular membranes, declined from 72.43% in the control to 39.05% under drought, indicating severe membrane damage. Similarly, higher LMSI values indicate better protection against stress-induced membrane damage, thereby supporting normal physiological and metabolic processes under drought conditions. Single inoculations significantly improved LMSI to 49.87% (AC), 55.21% (PP), and 50.46% (BS), corresponding to increases of 27.7%, 41.4%, and 29.3%, respectively, over drought. The combined consortium restored LMSI to 64.98%, reflecting a 66.4% improvement relative to drought and exceeding individual treatments by 17.7–30.4%.

Lipid peroxidation, measured as malondialdehyde (MDA) content, increased from 0.72 nmol g⁻¹ FW in the control to 2.36 nmol g⁻¹ FW under drought, representing a 227.8% increase. Inoculation with *(A) chroococum*, *P. putida*, and *(B) subtilis* reduced MDA levels to 1.63, 1.28, and 1.53 nmol g⁻¹ FW, corresponding to reductions of 30.9%, 45.8%, and 35.2%, respectively, compared with drought. The consortium resulted in the lowest MDA content (1.13 nmol g⁻¹ FW), showing a 52.1% reduction relative to drought and a 11.7–24.5% decrease compared with individual bacterial treatments.

### Photosynthetic pigments and osmotic adjustment

**Fig. 2:**
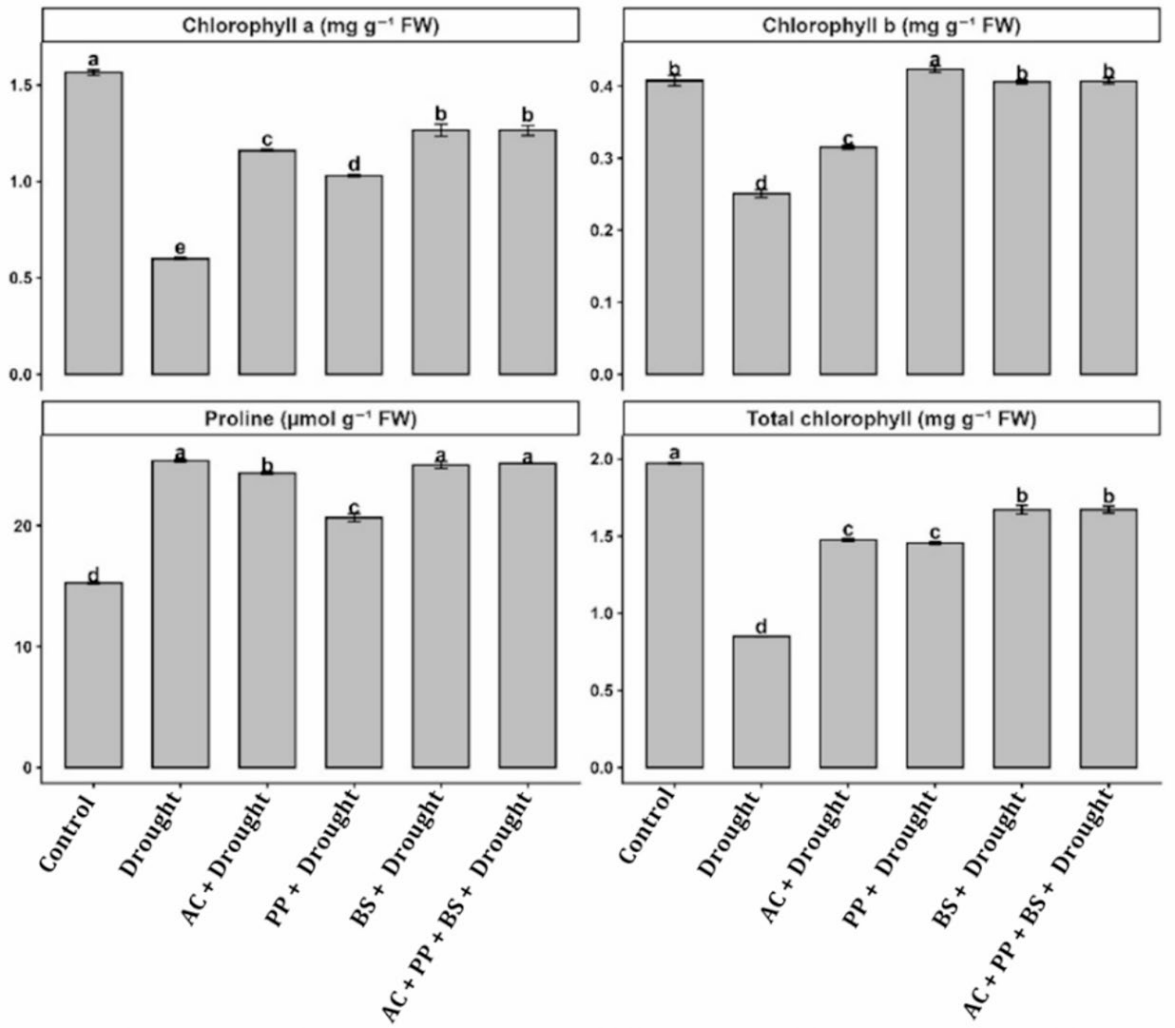
Effect of rhizobacterial application on (**A**) chlorophyll a, (**B**) chlorophyll b, (**C**) proline content, and (**D**) total chlorophyll of tomato under drought stress. Bars represent mean values ± standard error (SE) of three biological replicates (*n* = 3). Different lowercase letters above the bars indicate significant differences among treatments according to the LSD test at *p* ≤ 0.05.

Drought stress caused a pronounced decline in photosynthetic pigments (Fig. 2). Chlorophyll a content decreased from 1.56 mg g⁻¹ FW in the control to 0.58 mg g⁻¹ FW under drought, representing a 62.8% reduction. Inoculation with *(A) chroococum* increased chlorophyll a to 1.18 mg g⁻¹ FW (103.4% increase over drought), while P. putida and (B) subtilis increased it to 1.05 mg g⁻¹ FW (81.0%) and 1.28 mg g⁻¹ FW (120.7%), respectively. The consortium recorded the highest chlorophyll a content (1.31 mg g⁻¹ FW), reflecting a 125.9% increase compared with drought and exceeding individual inoculations by 2.3–24.8%. Chlorophyll b content declined by 44.2% under drought relative to the control. Single inoculations partially restored chlorophyll b, whereas consortium-maintained values (0.43 mg g⁻¹ FW) were statistically comparable to the control and 34–38% higher than in drought-stressed plants.

Total chlorophyll content decreased from 1.98 mg g⁻¹ FW in the control to 0.85 mg g⁻¹ FW under drought, representing a 57.1% reduction. *(A) chroococum*, *P. putida*, and *(B) subtilis* increased total chlorophyll to 1.47, 1.44, and 1.68 mg g⁻¹ FW, corresponding to 72.9%, 69.4%, and 97.6% increases, respectively, over drought. The consortium achieved the highest total chlorophyll content (1.70 mg g⁻¹ FW), a 100.0% increase relative to drought, and surpassed individual treatments by 1.2–18.1%.

Proline content increased from 15.2 µmol g⁻¹ FW in the control to 26.1 µmol g⁻¹ FW under drought, corresponding to a 71.7% increase. Inoculation with *(A) chroococum*,* P. putida*, and *(B) subtilis* resulted in proline contents of 24.6, 21.1, and 25.2 µmol g⁻¹ FW, respectively. The consortium maintained high proline accumulation (25.6 µmol g⁻¹ FW), representing a 68.4% increase over the control, comparable to the most effective single inoculations.

### Antioxidant enzyme responses to rhizobacteria under drought stress

**Fig. 3:**
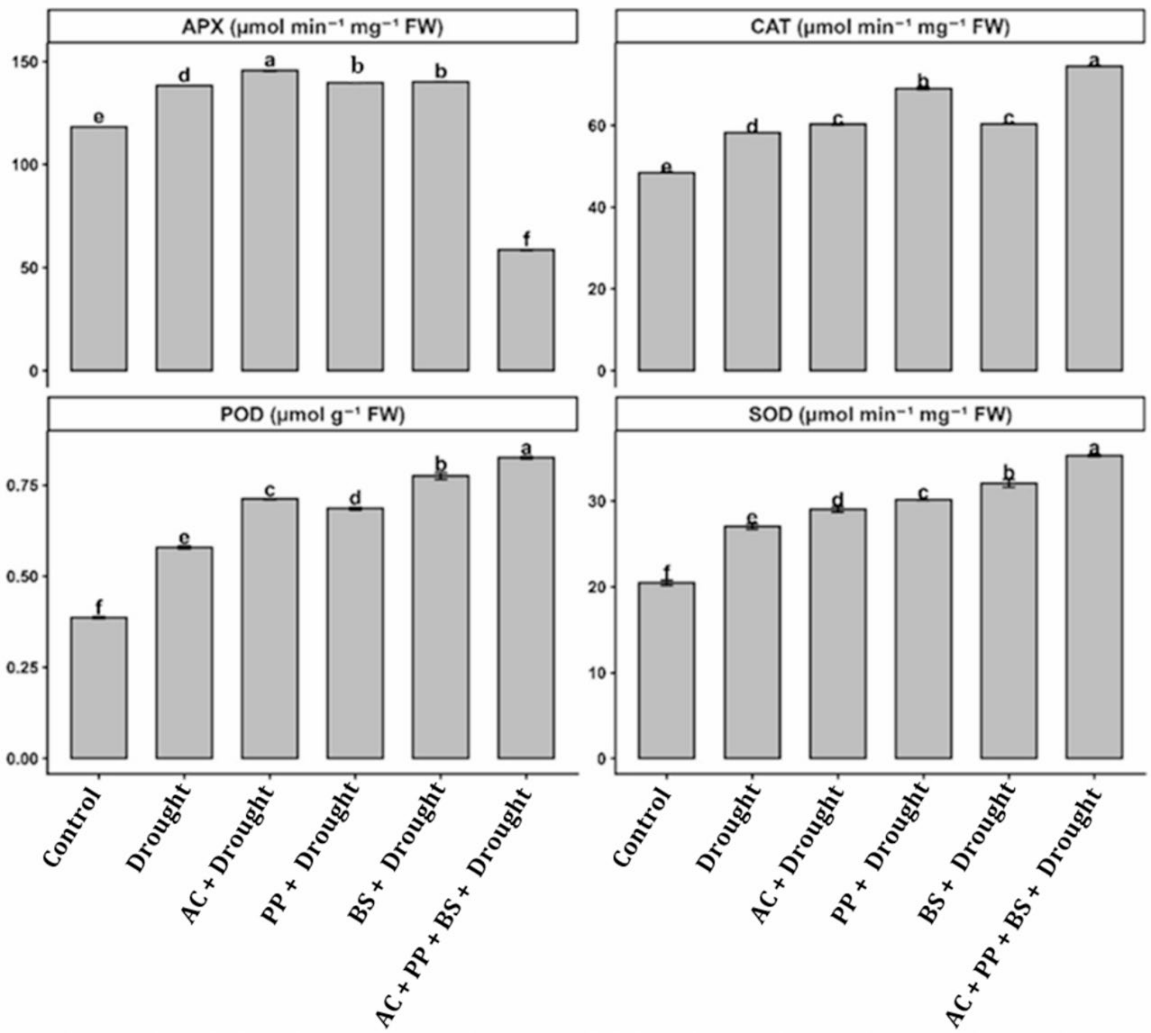
Effect of rhizobacterial application on the activities of antioxidant enzymes in tomato under drought stress. (**A**) ascorbate peroxidase (APX, µmol min⁻¹ mg⁻¹ FW), (**B**) catalase (CAT, µmol min⁻¹ mg⁻¹ FW), (**C**) peroxidase (POD, µmol g⁻¹ FW) and (**D**) superoxide dismutase (SOD, µmol min⁻¹ mg⁻¹ FW). Bars represent mean values ± standard error (SE), *n* = 3. Different lowercase letters indicate significant differences among treatments according to the LSD test at *p* ≤ 0.05.

Drought stress significantly altered the antioxidant defense system of tomato plants (Fig. 3), as reflected by changes in the activities of superoxide dismutase (SOD), ascorbate peroxidase (APX), catalase (CAT), and peroxidase (POD) (Fig. 3). Compared with the control, drought stress induced a moderate increase in antioxidant enzyme activities; however, rhizobacterial inoculation markedly amplified these responses, with the combined consortium AC + PP+BS consistently exhibiting the highest enzymatic activities.

Superoxide dismutase (SOD) activity increased from 20.6 µmol min⁻¹ mg⁻¹ FW in the control to 27.3 µmol min⁻¹ mg⁻¹ FW under drought, corresponding to a 32.5% increase. Inoculation with *Azotobacter chroococum* further enhanced SOD activity to 29.4 µmol min⁻¹ mg⁻¹ FW, representing a 7.7% increase over drought. *Pseudomonas putida* and *Bacillus subtilis* resulted in SOD activities of 30.1 and 32.2 µmol min⁻¹ mg⁻¹ FW, corresponding to 10.3% and 17.9% increases, respectively, compared with drought. The consortium recorded the highest SOD activity (35.4 µmol min⁻¹ mg⁻¹ FW), reflecting a 29.7% increase over drought and exceeding individual inoculations by 10.0–20.4%.

Ascorbate peroxidase (APX) activity increased from 118.2 µmol min⁻¹ mg⁻¹ FW in the control to 138.6 µmol min⁻¹ mg⁻¹ FW under drought, corresponding to a 17.3% increase. Application of *(A) chroococum* significantly increased APX activity to 147.9 µmol min⁻¹ mg⁻¹ FW, representing a 6.7% increase over the drought. *P. putida* and *(B) subtilis* showed APX activities of 141.3 and 144.2 µmol min⁻¹ mg⁻¹ FW, corresponding to 2.0% and 4.0% increases, respectively, compared with drought. In contrast, the consortium showed a distinctly higher APX activity (159.8 µmol min⁻¹ mg⁻¹ FW), representing a 15.3% increase over drought and a 7.5–13.1% increase compared with individual inoculations.

Catalase (CAT) activity increased from 48.7 µmol min⁻¹ mg⁻¹ FW in the control to 58.6 µmol min⁻¹ mg⁻¹ FW under drought, corresponding to a 20.3% increase. Inoculation with *(A) chroococum* increased CAT activity to 60.9 µmol min⁻¹ mg⁻¹ FW (3.9% over drought), while *P. putida* and *(B) subtilis* resulted in activities of 68.7 and 61.2 µmol min⁻¹ mg⁻¹ FW, corresponding to 17.2% and 4.4% increases, respectively. The consortium exhibited the highest CAT activity (74.6 µmol min⁻¹ mg⁻¹ FW), reflecting a 27.3% increase over drought and exceeding individual treatments by 8.6–22.5%.

Peroxidase (POD) activity showed the strongest relative response to rhizobacterial inoculation. POD activity increased from 0.42 µmol g⁻¹ FW in the control to 0.56 µmol g⁻¹ FW under drought, representing a 33.3% increase. Single inoculations with *(A) chroococum*, *P. putida*, and *(B) subtilis* increased POD activity to 0.72, 0.65, and 0.81 µmol g⁻¹ FW, corresponding to increases of 28.6%, 16.1%, and 44.6%, respectively, over drought. The consortium achieved the highest POD activity (0.89 µmol g⁻¹ FW), representing a 58.9% increase over drought and exceeding individual inoculations by 9.9–36.9%.

### Growth and morphological responses of tomato under drought stress

**Fig. 4:**
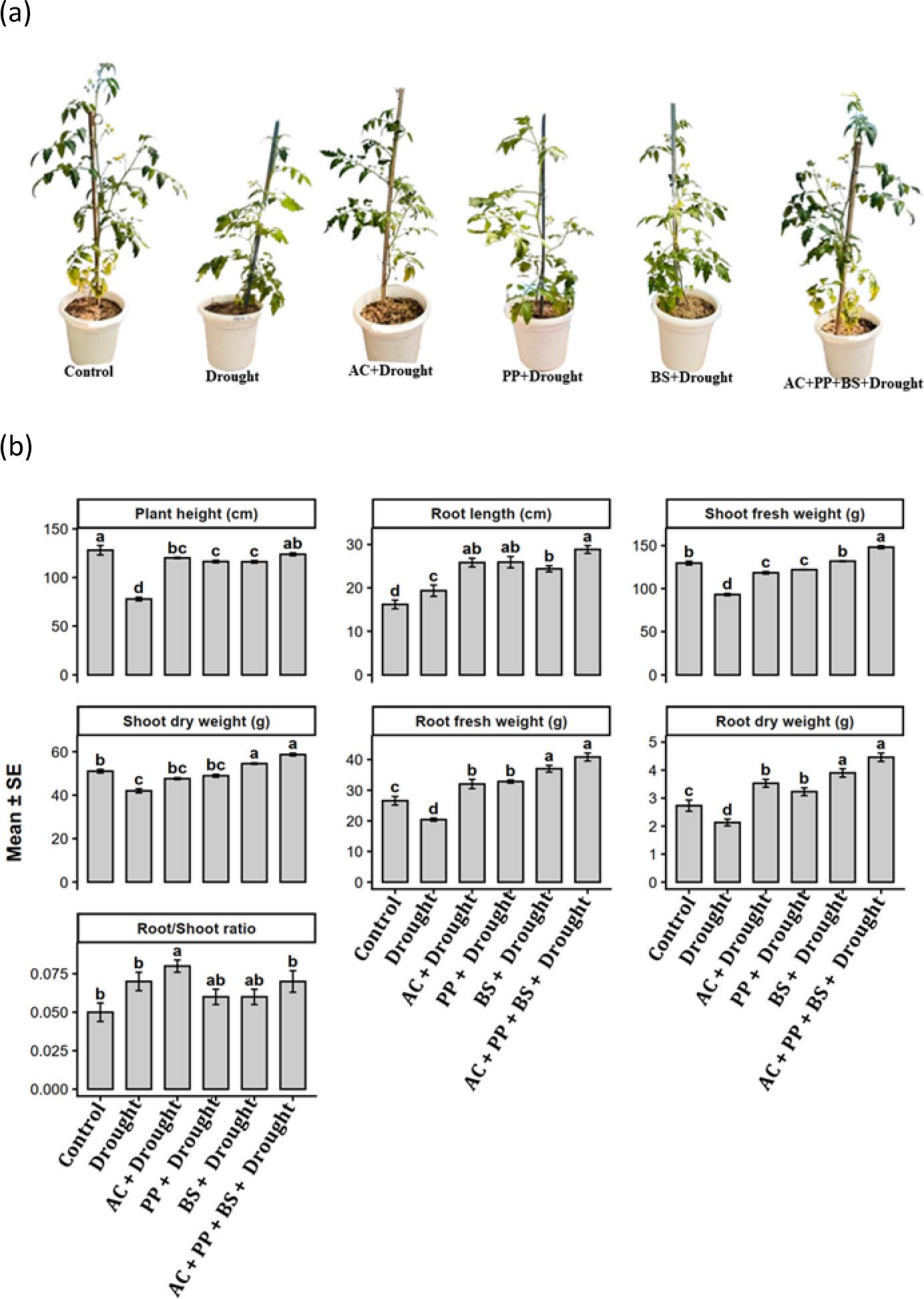
(**a**) Effect of single and combined rhizobacterial inoculation on the growth performance of tomato plants under drought stress. (**b**) Effect of rhizobacterial treatments on growth and biomass attributes of tomato under drought stress. Mean (± SE) values of plant height, root length, shoot fresh weight, shoot dry weight, root fresh weight, root dry weight, and root/shoot ratio of tomato plants subjected to different rhizobacterial treatments under drought stress. Bars represent treatment means based on three replications (*n* = 3). Different lowercase letters above bars indicate significant differences among treatments according to the LSD test at *p* ≤ 0.05.

Drought stress significantly impaired the growth and morphological performance of tomato plants (Fig. 4a and b). Plant height declined from 128.00 cm in the control to 77.74 cm under drought, corresponding to a 39.3% reduction. Inoculation with *Azotobacter chroococum* (AC) under drought markedly improved plant height to 120.12 cm, representing a 54.4% increase over drought-stressed plants. Similarly, *Pseudomonas putida* (PP) and *Bacillus subtilis* (BS) increased plant height to 116.34 cm and 116.12 cm, corresponding to increases of 49.6% and 49.3%, respectively, relative to drought. The combined consortium AC + PP+BS resulted in the highest plant height (123.82 cm), reflecting a 59.3% improvement over drought and exceeding individual inoculations by 3.1–6.6%.

Root length increased slightly under drought stress (19.35 cm) compared with the control (16.18 cm). However, rhizobacterial inoculation substantially enhanced root elongation. AC, PP, and BS increased root length to 25.83 cm, 25.93 cm, and 24.40 cm, corresponding to increases of 33.5%, 34.0%, and 26.1%, respectively, over the drought. The consortium recorded the highest root length (28.86 cm), representing a 49.1% increase over drought and surpassing individual inoculants by 11.3–18.3%.

Shoot fresh weight declined from 129.60 g in the control to 93.06 g under drought, indicating a 28.2% reduction. Application of AC, PP, and BS increased shoot fresh weight to 118.37 g, 121.93 g, and 131.83 g, corresponding to increases of 27.2%, 31.0%, and 41.7%, respectively, over the drought. The consortium exhibited the highest shoot fresh weight (148.10 g), reflecting a 59.2% increase compared with drought and exceeding the best single inoculant (BS) by 12.3%. A similar trend was observed for shoot dry weight, which declined by 17.7% under drought relative to the control. AC, PP, and BS increased shoot dry weight to 47.60 g, 48.93 g, and 54.53 g, representing increases of 13.3%, 16.5%, and 29.8%, respectively, over the drought. The consortium recorded the highest shoot dry weight (58.70 g), corresponding to a 39.8% increase over drought and a 7.6–23.3% improvement over individual inoculations.

Root fresh weight decreased from 26.56 g in the control to 20.40 g under drought, representing a 23.2% reduction. Inoculation with AC, PP, and BS increased root fresh weight to 32.03 g, 32.83 g, and 37.00 g, corresponding to increases of 57.0%, 60.9%, and 81.4%, respectively, over drought. The consortium exhibited the highest root fresh weight (40.83 g), reflecting a 100.1% increase relative to drought and exceeding individual inoculants by 10.3–27.6%. Root dry weight followed a comparable pattern, declining from 2.73 g in the control to 2.13 g under drought (22.0% reduction). AC, PP, and BS increased root dry weight to 3.53 g, 3.23 g, and 3.90 g, corresponding to increases of 65.7%, 51.6%, and 83.1%, respectively, over drought. The consortium resulted in the maximum root dry weight (4.46 g), representing a 109.4% increase relative to drought and exceeding individual treatments by 14.4–38.1%. The root to shoot ratio increased under drought stress (0.07) compared with the control (0.05). AC further increased the ratio to 0.08, while PP, BS, and the consortium s-maintained values (0.06–0.07) statistically comparable to drought-stressed plants.

### Yield and yield-related traits of tomato under drought stress

**Fig. 5:**
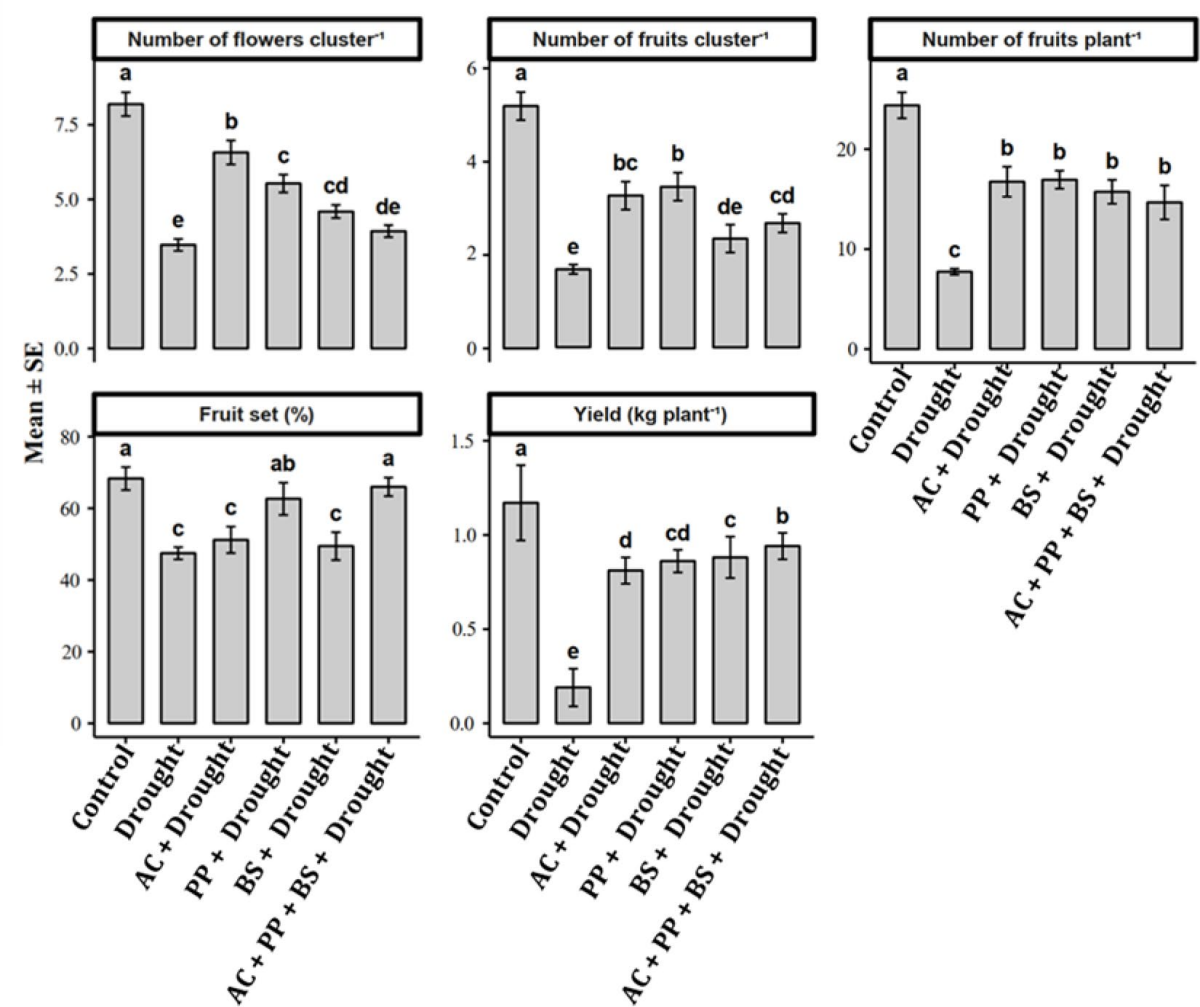
Influence of rhizobacterial inoculation on flowering, fruiting, and yield attributes of tomato under drought stress. Bars represent mean ± standard error (SE) of three replications (*n* = 3). Different lowercase letters above bars indicate significant differences among treatments according to the LSD test at *p* ≤ 0.05.

Drought stress caused a pronounced decline in tomato plant reproductive performance and yield (Fig. 5). The number of flowers per cluster decreased from 8.19 under the control to 3.47 under drought, corresponding to a 57.6% reduction. Inoculation with *Azotobacter chroococum* (AC) significantly increased the number of flowers to 6.57, representing an 89.3% improvement over the drought. *Pseudomonas putida* (PP) and *Bacillus subtilis* (BS) increased flower numbers to 5.53 and 4.59, corresponding to 59.4% and 32.3% increases, respectively, relative to drought. The consortium (AC + PP+BS) recorded 3.93 flowers per cluster, representing a 13.3% increase over drought, but remaining lower than individual inoculations.

A similar trend was observed for the number of fruits per cluster. Drought stress reduced fruit number from 5.19 in the control to 1.67, reflecting a 67.8% decline. AC, PP, and BS increased fruits per cluster to 3.27, 3.46, and 2.33, corresponding to increases of 95.8%, 107.2%, and 39.5% over drought, respectively. The consortium resulted in 2.66 fruits per cluster, a 59.3% increase over drought, although values remained lower than those recorded for the AC and PP.

The number of fruits per plant was severely reduced by drought, declining from 24.39 in the control to 7.73, corresponding to a 68.3% reduction. Rhizobacterial inoculation markedly improved fruit number. AC, PP, and BS increased fruit number to 16.73, 16.93, and 15.72 fruits per plant, corresponding to increases of 116.4%, 119.0%, and 103.4%, respectively, over drought. The consortium produced 14.66 fruits per plant, representing a 89.7% increase relative to drought, and the values were statistically comparable to those of individual inoculants.

Fruit set percentage declined significantly under drought stress, decreasing from 68.27% in the control to 47.43%, corresponding to a 30.5% reduction. AC and BS increased fruit set to 51.15% and 49.42%, representing gains of 7.8% and 4.2%, respectively, relative to drought stress. PP further improved fruit set to 62.65%, a 32.1% increase compared with drought conditions. The consortium produced the highest fruit set (65.99%), representing a 39.1% improvement and restoring levels to near those of the control.

Drought stress caused a pronounced reduction in tomato yield, with per-plant yield declining from 1.17 kg under well-watered conditions to 0.19 kg under drought stress, representing an 83.8% reduction. In contrast, all rhizobacterial treatments significantly improved yield under drought conditions. Plants inoculated with *Azotobacter chroococcum*, *Pseudomonas putida*, and *Bacillus subtilis* produced yields of 0.81, 0.86, and 0.88 kg plant⁻¹, respectively, indicating substantial recovery of yield compared with drought-stressed plants. The combined rhizobacterial consortium achieved the highest yield (0.94 kg plant⁻¹), exceeding yields from individual inoculations by 6.8–16.0%, demonstrating the superiority of the combined microbial approach over single-strain applications.

### Coordinated physiological and biochemical responses shaping growth and yield under drought stress

**Fig. 6:**
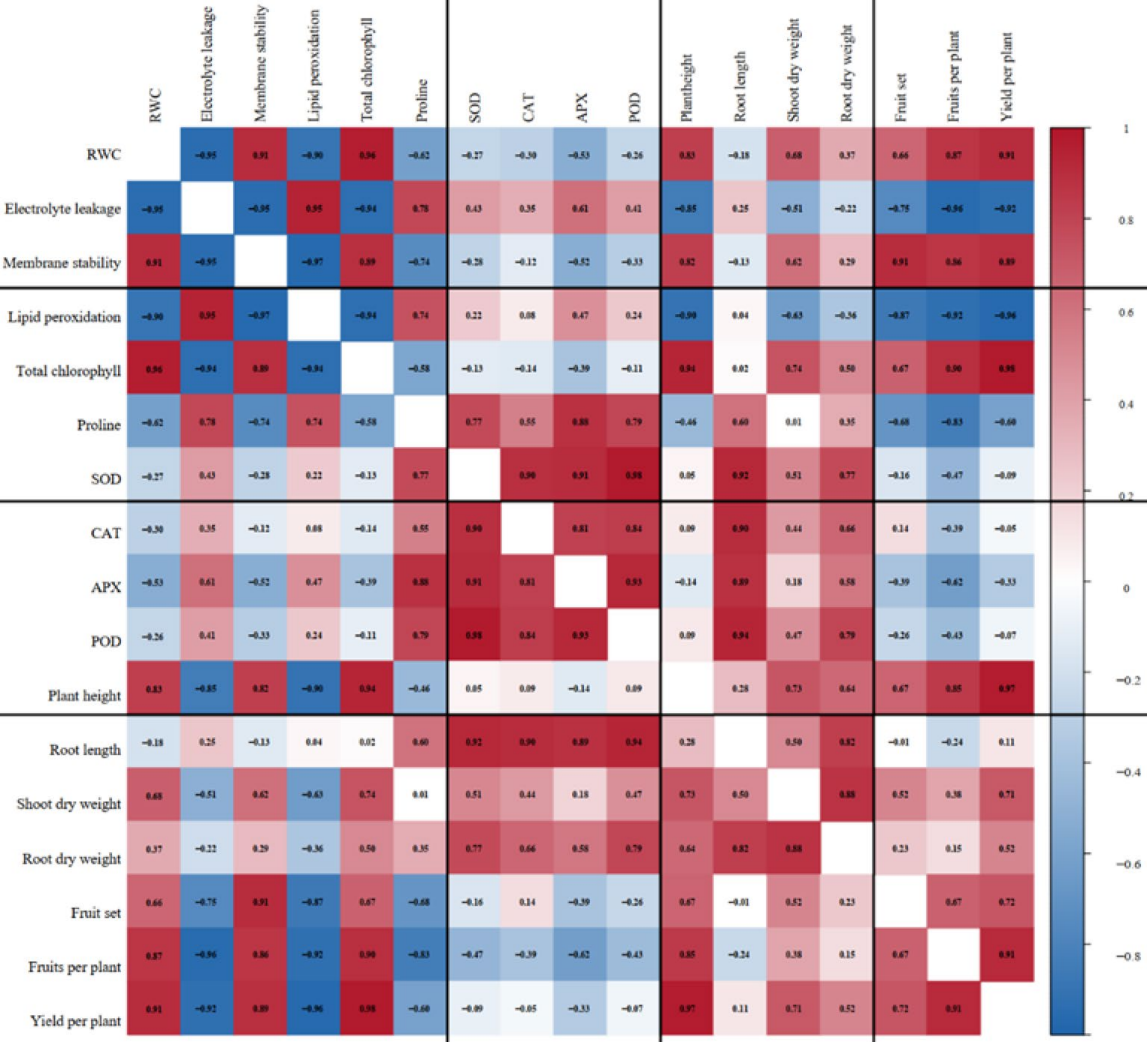
Correlation matrix depicting relationships among physiological, biochemical, growth, and yield traits of tomato under drought stress. Pearson’s correlation heatmap showing the relationships among measured physiological, biochemical, growth, and yield attributes of tomato under drought conditions. The colour gradient represents the strength and direction of correlations, ranging from strong negative (blue) to strong positive (red), with numerical correlation coefficients shown within each cell. Bold values indicate statistically significant correlations (*p* ≤ 0.05). Black demarcation lines separate different attribute groups to improve visual interpretation.

The correlation heatmap revealed strong and statistically significant associations among physiological traits, antioxidant responses, morphological attributes, and yield components under drought stress (Fig. 6). Relative water content (RWC) was strongly and positively correlated with total chlorophyll content (*r* = 0.96) and yield per plant (*r* = 0.91). In contrast, it was strongly negatively correlated with electrolyte leakage (*r* = − 0.95) and lipid peroxidation (*r* = − 0.90). A similar pattern was observed for membrane stability, which exhibited significant positive relationships with total chlorophyll (*r* = 0.89), fruit set (*r* = 0.91), fruits per plant (*r* = 0.86), and yield per plant (*r* = 0.89), alongside a pronounced negative association with lipid peroxidation (*r* = − 0.97). In contrast, electrolyte leakage and lipid peroxidation were both strongly and negatively correlated with yield per plant (*r* = − 0.92 and − 0.96, respectively), indicating that oxidative and membrane damage were closely linked to yield losses under drought conditions.

Photosynthetic capacity emerged as a central determinant of plant productivity under water deficit. Total chlorophyll content showed strong positive correlations with plant height (*r* = 0.94), shoot dry weight (*r* = 0.74), fruits per plant (*r* = 0.90), and yield per plant (*r* = 0.98), highlighting the importance of preserving chlorophyll to sustain growth and yield under drought stress. In contrast, proline accumulation was positively associated with antioxidant enzyme activities, including superoxide dismutase (SOD; *r* = 0.77), catalase (CAT; *r* = 0.55), ascorbate peroxidase (APX; *r* = 0.88), and peroxidase (POD; *r* = 0.79), but showed significant negative relationships with fruits per plant (*r* = − 0.83) and yield per plant (*r* = − 0.60). This pattern suggests that proline accumulation primarily reflected stress perception and protective adjustment rather than direct yield enhancement.

Antioxidant enzymes formed a tightly coordinated response network, as indicated by strong positive correlations among SOD, CAT, APX, and POD activities. SOD activity was strongly correlated with CAT (*r* = 0.90), APX (*r* = 0.91), and POD (*r* = 0.98), while CAT also exhibited strong associations with APX (*r* = 0.81) and POD (*r* = 0.84). These antioxidant enzymes were positively linked with root growth traits, particularly root length (SOD: *r* = 0.92; CAT: *r* = 0.90; APX: *r* = 0.89; POD: *r* = 0.94), indicating enhanced antioxidant-mediated support for below-ground development under drought stress. However, antioxidant activities showed weak to moderate negative correlations with yield per plant (*r* = − 0.09 to − 0.33), reinforcing their primary role in stress mitigation rather than in directly determining yield.

Morphological traits exhibited strong and consistent associations with yield performance. Plant height was positively correlated with fruit set (*r* = 0.67), fruits per plant (*r* = 0.85), and yield per plant (*r* = 0.97). Shoot dry weight also showed positive relationships with fruits per plant (*r* = 0.38) and yield per plant (*r* = 0.71), while root dry weight was moderately but significantly associated with yield per plant (*r* = 0.52). Yield components themselves were closely interrelated, with fruits per plant showing a strong positive correlation with yield per plant (*r* = 0.91) and fruit set (*r* = 0.67).

Collectively, these correlations delineate a clear physiological–biochemical–morphological continuum governing yield performance under drought stress. Traits related to improved water status, membrane stability, and photosynthetic capacity were tightly coupled with yield attributes, whereas indicators of oxidative damage exhibited strong antagonistic relationships. The present study demonstrated a significant enhancement in antioxidant enzyme activities with rhizobacterial treatments, indicating improved regulation of oxidative stress under drought conditions. This was further supported by consistent trends in oxidative damage indicators, including reduced malondialdehyde content, lower electrolyte leakage, and improved membrane stability. These responses collectively suggest effective mitigation of oxidative stress in treated plants. Further investigation of reactive oxygen species dynamics would provide additional insights into antioxidant-mediated drought-tolerance mechanisms.

### Comparative performance of rhizobacterial treatments under drought stress

**Fig. 7:**
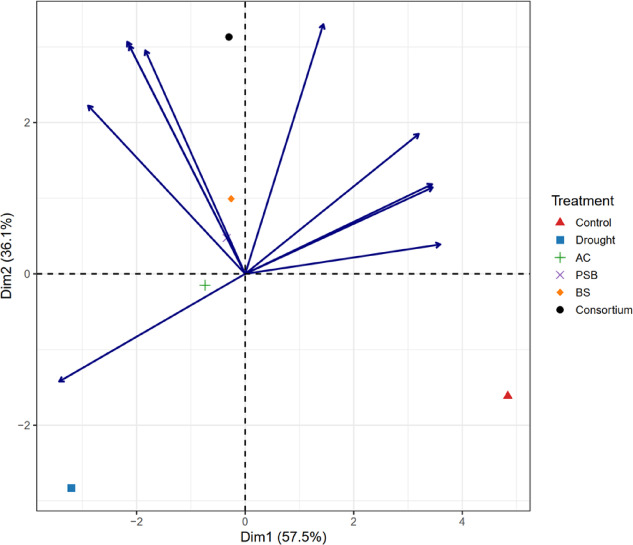
Principal component analysis of rhizobacterial treatments under drought stress. Principal component analysis (PCA) biplot illustrating the multivariate relationships among rhizobacterial treatments and the measured attributes under drought conditions. Points represent treatments, while vectors indicate the direction and relative contribution of the evaluated characteristics to the principal component space. The spatial separation and proximity of treatments reflect differences and similarities in their overall response to drought stress.

Principal component analysis (PCA) was used to integrate physiological, biochemical, morphological, and yield-related traits and to compare the overall performance of rhizobacterial treatments under drought stress (Fig. 7). The first two principal components accounted for most of the variation among treatments: PC1, 57.5%; PC2, 36.1%; for a total of 93.6%.

PC1 mainly represented overall plant performance under drought stress. It showed strong positive associations with yield per plant, fruits per plant, fruit set, plant height, shoot dry weight, total chlorophyll content, and relative water content, while showing negative associations with electrolyte leakage and lipid peroxidation. Treatments located on the positive side of PC1 were therefore characterized by better water status, lower oxidative damage, improved growth, and higher yield. In contrast, drought-stressed plants without microbial inoculation clustered on the negative side of PC1, indicating poor physiological condition and reduced productivity.

PC2 primarily reflected biochemical stress responses. This component was positively associated with antioxidant enzyme activities (SOD, CAT, APX, and POD) and proline accumulation. Treatments positioned higher along PC2 showed stronger activation of antioxidant defense mechanisms, suggesting a greater capacity to mitigate drought-induced oxidative stress.

Clear separation among treatments was evident in the PCA biplot. The rhizobacterial consortium was distinctly positioned in the positive region of both PC1 and PC2 and closely aligned with yield-, biomass-, and chlorophyll-related traits along PC1. In contrast, antioxidant enzymes were primarily associated with PC2, representing biochemical stress-response mechanisms. This separation indicates that antioxidant responses are primarily involved in stress mitigation rather than directly contributing to yield, which is consistent with the negative correlations observed between antioxidant enzymes and yield. This distribution indicates that the consortium promoted a well-coordinated response, combining improved physiological status, enhanced biochemical defense, and sustained growth under drought stress. Among the single inoculants, *Bacillus subtilis* showed comparatively better performance, occupying an intermediate position closer to growth- and yield-related traits. In contrast, *Pseudomonas putida* and *Azotobacter chroococcum* were positioned nearer to the origin, indicating moderate improvement over drought stress alone.

The well-watered control was located on the positive side of PC1. Still, it was separated from antioxidant-related vectors, reflecting optimal growth and yield under non-stress conditions with limited activation of stress-defense pathways. In contrast, the drought treatment clustered in the negative region of both PC1 and PC2 and was closely associated with indicators of oxidative damage, confirming its poor overall performance. Among drought-stressed treatments, the rhizobacterial consortium showed the strongest positive displacement along both principal components, demonstrating its superior ability to integrate physiological protection, antioxidant defense, and growth processes to sustain yield under drought conditions compared with individual inoculants.

## Discussion

The combined application of all three rhizobacterial strains significantly increased plant height, root length, and root and shoot biomass, consistent with the established role of plant growth-promoting rhizobacteria (PGPR) in mitigating drought stress. While the present study demonstrated clear improvements in plant growth, physiological performance, and biochemical responses following rhizobacterial inoculation, direct assessment of rhizosphere colonization by the applied strains was not undertaken. Therefore, the observed responses should be interpreted as indicative of functional associations between inoculation and plant performance. Future studies employing microbial tracking techniques, such as marker-based or molecular approaches, would help to confirm colonization dynamics and establish stronger causal relationships. Research highlights the protective effects of ACC deaminase-producing PGPR, such as *Bacillus subtilis*, against drought-induced oxidative damage, thereby enhancing plant growth^28^. Similarly, Vurukonda et al.^4^ emphasize that PGPR facilitate plant growth under stress through mechanisms like modulating phytohormone levels and improving nutrient uptake. The observed increases in root and shoot biomass in this study are particularly noteworthy, as a strong root system is essential for effective water acquisition under drought conditions. Mayak et al.^27^ underscore the pivotal role of PGPR in promoting root growth, a finding echoed in your results. Enhanced shoot biomass, indicative of improved plant vigor and photosynthetic capacity, further highlights the potential for greater yield under stress conditions.

The synergistic effects observed in the combined treatment suggest complementary interactions among the rhizobacteria, leading to amplified benefits for plant growth and stress tolerance. Each strain contributes distinct functional attributes; *Azotobacter* enhances nitrogen fixation, *Pseudomonas* supports phosphate solubilization and produces growth-promoting substances, while *Bacillus* improves nutrient availability and stress resilience^4,9,14,17^. Together, these rhizobacteria likely optimize nutrient acquisition and activate stress-response pathways, resulting in the observed improvements, as reported in previous studies on microbial consortia under drought stress^4,9^. For example, Subramanian et al.^29^ demonstrated the positive effects of arbuscular mycorrhizal fungi on nutrient uptake and yield under drought stress, a concept echoed in the enhanced resource acquisition facilitated by rhizobacteria in your study. Furthermore, Liang et al.^30^ underscore the importance of understanding tomato’s photosynthetic and physiological responses to drought, and your work contributes by showing how PGPR mitigate these adverse effects.

The study also reported that the combined application of all three rhizobacteria strains significantly boosted the number of flowers per cluster and fruits per plant, highlighting their potential to enhance plant reproductive development. The observed increase is likely due to improved plant health and nutrient availability. Gen-Jiménez et al.^26^ demonstrated that Rhizobium biofortification can enhance nutrient uptake and overall plant growth, indirectly supporting increased flowering. The most notable outcome of the combined treatment is the marked increase in yield per plant. This result underscores the practical potential of using these rhizobacteria to improve tomato production under drought conditions. The higher fruit setting percentage further illustrates the treatment’s positive impact on reproductive success. Ruzzi and Aroca^25^ highlight the role of PGPR as bio-stimulants in horticulture, emphasizing their ability to boost yield and stress tolerance.

Drought stress significantly reduced the relative water content (RWC) of tomato plants, reflecting dehydration, a key consequence of water deficit that disrupts cellular functions and impairs metabolic processes^31^. Treatments with individual rhizobacteria partially mitigated this effect, likely by improving root development and water uptake^27^. Among these, the combined AC + PP+BS showed the most significant improvement, restoring RWC to levels closer to those of well-watered plants and suggesting an enhanced ability to retain cellular hydration under drought conditions. Drought also triggered a marked increase in malondialdehyde (MDA) levels, a clear indicator of oxidative stress^32^. All rhizobacteria significantly reduced MDA content, with the combined treatment showing the most pronounced effect. This reduction suggests enhanced protection against oxidative damage, possibly due to improved antioxidant activity, such as increased production of antioxidant enzymes or the scavenging of reactive oxygen species^28^.

Electrolyte leakage, an indicator of cell membrane damage, rose sharply under drought conditions. However, rhizobacteria effectively reduced this leakage, particularly the combined treatment, which also enhanced the membrane stability index. Maintaining membrane integrity is critical under stress, as it preserves membrane selectivity and prevents the loss of essential ions and metabolites^33^. Rhizobacteria may aid this by modifying lipid composition and reducing oxidative damage to membrane lipids^32^. Drought negatively affected photosynthetic pigments, reducing chlorophyll a, chlorophyll b, and total chlorophyll content. However, rhizobacteria, especially the combined treatment, significantly increased these pigments, indicating enhanced photosynthetic capacity under stress. This could be due to improved nutrient uptake, particularly nitrogen^26^, or better protection of the photosynthetic apparatus from oxidative damage^30^. Additionally, proline accumulation, a key osmo-protectant, increased under drought stress and was further enhanced by the combined treatment. This suggests improved osmotic stress tolerance, possibly through rhizobacteria-induced proline biosynthesis or reduced degradation^25,34^.

The combined AC + PP+BS consistently outperformed individual applications across all parameters, underscoring the synergistic interactions among these rhizobacteria. Azotobacter contributes nitrogen fixation, Pseudomonas promotes growth and systemic resistance, and Bacillus produces beneficial metabolites^4^. Together, these strains likely optimize physiological processes, including nutrient availability, hormone regulation, and the production of stress-protective compounds, thereby enhancing drought tolerance^35^.

The findings of this study demonstrate the significant influence of rhizobacteria, particularly the combined application of *Azotobacter chroococum*, *Pseudomonas putida*, and *Bacillus subtilis*, on the antioxidant enzyme activities of tomato plants subjected to drought stress. These enzymes are crucial for mitigating oxidative damage by detoxifying reactive oxygen species (ROS), which are produced in excess under drought conditions and can harm cellular components. The combined rhizobacteria consistently outperformed individual treatments and the control, significantly enhancing the activities of superoxide dismutase (SOD), ascorbate peroxidase (APX), catalase (CAT), and peroxidase (POD). This improvement highlights the synergistic interactions among the three rhizobacteria strains, resulting in a more robust antioxidant defense system in the tomato plants.

SOD activity, which catalyzes the conversion of superoxide radicals into hydrogen peroxide and oxygen, increased substantially with the combined treatment compared to the control. This highlights the treatment’s enhanced capacity to neutralize superoxide radicals and minimize oxidative damage, consistent with previous studies showing that plant growth-promoting rhizobacteria (PGPR) increase SOD activity under drought stress^33,36^. Similarly, APX, which plays a central role in detoxifying hydrogen peroxide within the ascorbate-glutathione cycle, showed increased activity with the combined treatment. This suggests a more efficient mechanism for hydrogen peroxide detoxification, providing further protection against oxidative stress. These findings align with reports of increased APX activity in plants treated with beneficial microbes during drought conditions^37,38^. The relatively moderate response of APX activity under single-strain inoculation, compared with its pronounced increase under the combined consortium, may be attributed to synergistic interactions among the rhizobacterial strains. Individual strains may induce only partial activation of antioxidant pathways, whereas the consortium likely triggers a more coordinated and amplified response through complementary mechanisms, including improved nutrient acquisition, enhanced phytohormone signaling, and stronger root-microbe interactions. This combined effect may reach a threshold level of oxidative signaling that activates APX more effectively, thereby enhancing the detoxification of hydrogen peroxide under drought stress^39^.CAT activity, which directly converts hydrogen peroxide into water and oxygen, increased in the combined treatment. This provides an alternative and complementary pathway for hydrogen peroxide detoxification, reinforcing the antioxidant defense system. Such enhancement of CAT activity by PGPR under drought and other stress conditions has been documented in earlier research^40^. POD activity, which aids in ROS detoxification and contributes to cell wall reinforcement, demonstrated the most dramatic increase, doubling in the combined treatment compared to the control. This suggests a multifaceted role for POD in mitigating stress, consistent with evidence showing rhizobacteria-induced increases in POD activity under drought stress^27,28^. The synergistic enhancement of these antioxidant enzymes in the combined treatment can be attributed to several factors, including improved nutrient availability, increased phytohormone production, and enhanced synthesis of stress-protective compounds. The combination of AC, PP, and BS likely fosters a more favorable rhizosphere environment, promoting plant growth and resilience under drought conditions. These findings underscore the potential of such rhizobacterial consortia to strengthen plant defense mechanisms and improve stress tolerance.

The correlation analysis revealed that drought tolerance and yield performance in tomato were governed by an integrated network of physiological, biochemical, and morphological processes rather than by individual traits acting in isolation. The strong positive association between relative water content (RWC) and total chlorophyll content, together with its close relationship with per-plant yield, highlights the central role of plant water status in sustaining photosynthetic efficiency and productivity under drought stress. Maintaining higher RWC under water-limited conditions enables continued stomatal regulation, preserves cellular hydration, and stabilizes metabolic processes, collectively supporting growth and yield formation. Similar relationships between RWC, chlorophyll retention, and yield stability under drought have been consistently reported in recent studies across diverse crop systems^41,42^.

The strong negative correlations between RWC, electrolyte leakage, and lipid peroxidation further underscore the importance of membrane integrity for drought tolerance. Electrolyte leakage and lipid peroxidation are widely recognized indicators of membrane damage caused by drought-induced oxidative stress. Their strong inverse association with yield per plant observed in the present study suggests that oxidative and membrane injuries are directly linked to productivity losses under water deficit. Plants that maintain membrane stability experience reduced ion leakage and lower lipid peroxidation, thereby preserving physiological function and growth potential. Recent investigations have similarly demonstrated that improved membrane stability under drought is closely associated with reduced oxidative damage and enhanced yield^43,44^.

Photosynthetic capacity emerged as a significant determinant of productivity under drought conditions, as evidenced by strong positive correlations with key growth and yield attributes, including plant height, shoot dry weight, fruit number per plant, and yield per plant. Chlorophyll preservation under drought stress ensures sustained light absorption and carbon assimilation, which are essential for maintaining biomass accumulation and reproductive success. Recent studies have emphasized that drought-tolerant plants often exhibit enhanced chlorophyll stability through improved water relations and antioxidant protection, leading to superior growth and yield under stressful environments^45,46^.

Proline accumulation showed a contrasting pattern, with strong positive associations with antioxidant enzyme activities and significant negative correlations with yield-related traits. This indicates that proline accumulation primarily reflected stress perception and activation of protective mechanisms rather than a direct contribution to yield enhancement. Proline is widely known to function as an osmo-protectant, redox buffer, and stabilizer of proteins and membranes during drought stress. However, increased proline levels are often associated with growth inhibition and metabolic adjustment toward survival rather than productivity. Recent literature supports the view that proline accumulation represents a stress-responsive indicator rather than a yield-promoting trait, particularly under severe or prolonged drought conditions^47,48^.

Antioxidant enzyme activities formed a highly coordinated cluster, as indicated by strong positive correlations among SOD, CAT, APX, and POD. This coordinated activation reflects an integrated antioxidant defense system that efficiently detoxifies reactive oxygen species generated during drought stress. Superoxide dismutase acts as the first line of defense by converting superoxide radicals into hydrogen peroxide, which CAT, APX, and POD subsequently scavenge. The synergistic functioning of antioxidant enzymes under drought has been widely reported as a key mechanism for maintaining cellular redox homeostasis and limiting oxidative damage^43,49^.

Notably, antioxidant enzyme activities showed strong positive correlations with root growth traits, particularly root length, suggesting that enhanced antioxidant protection supported root system development under drought conditions. A well-developed root system improves access to deeper soil moisture and enhances drought avoidance, thereby indirectly contributing to plant performance under stress. Recent studies have highlighted that antioxidant-mediated protection of root tissues is critical for sustaining root growth and function during water deficit^50,51^. However, the weak to moderate negative correlations between antioxidant activities and per-plant yield observed in this study indicate that antioxidant defense primarily supports stress mitigation and survival rather than directly driving yield formation. This pattern indicates that antioxidant enzymes primarily function in stress mitigation rather than directly contributing to yield formation, which is consistent with their separation from yield-related traits in the PCA analysis.

Morphological traits exhibited strong and consistent associations with yield components, reinforcing their integrative role in drought tolerance. Plant height and shoot dry weight were positively correlated with fruit set, fruits per plant, and yield per plant, reflecting the importance of sustained vegetative growth for reproductive success under drought stress. Root dry weight also showed a positive association with yield, emphasizing the contribution of below-ground biomass to water acquisition and yield stability. Similar relationships between morphological vigor and yield under drought have been reported in recent studies, highlighting the importance of balanced shoot and root growth for maintaining productivity under water-limited conditions^42,52^.

Overall, the correlation patterns observed in the present study delineate a clear physiological–biochemical–morphological continuum governing yield performance under drought stress. Traits associated with improved water status, membrane stability, and photosynthetic capacity were tightly coupled with yield attributes, whereas indicators of oxidative damage exhibited strong antagonistic relationships. These findings emphasize that drought tolerance in rhizobacteria-treated plants is achieved through coordinated regulation of multiple traits rather than isolated responses, underscoring the importance of integrated stress-adaptation mechanisms for sustaining growth and yield under drought.

The principal component analysis provided an integrated multivariate perspective on how rhizobacterial treatments modulated physiological, biochemical, morphological, and yield-related traits under drought stress. The high proportion of variance explained by the first two principal components indicates that drought-induced variability in plant performance was primarily governed by a limited set of tightly coordinated processes. The substantial contributions of yield attributes, chlorophyll content, relative water content, and growth traits to PC1 suggest that this axis primarily reflects overall physiological competence and productivity under water-limited conditions. Similar PCA-based interpretations have been reported in recent drought studies, in which PC1 typically represents a continuum from stress-induced damage to functional resilience and yield stability^42,52,53^.

The negative loading of electrolyte leakage and lipid peroxidation along PC1 reinforces the notion that membrane damage and oxidative stress significantly constrain productivity under drought. Treatments positioned toward the negative side of PC1 were therefore characterized by impaired cellular integrity and reduced growth. In contrast, those on the positive side exhibited improved water relations, preserved photosynthetic machinery, and enhanced yield formation. Recent studies have similarly demonstrated that PCA separation under drought is strongly driven by antagonistic relationships between membrane stability traits and yield-associated variables, highlighting the central role of oxidative damage in shaping stress responses^43,45^.

The second principal component captured variation associated with stress-responsive biochemical adjustments, particularly antioxidant enzyme activities and proline accumulation. The positive contributions of SOD, CAT, APX, POD, and proline to PC2 indicate that this axis reflects the intensity of stress perception and the activation of defensive metabolism. Antioxidant enzymes function as a coordinated network that mitigates drought-induced reactive oxygen species, while proline accumulation contributes to osmotic adjustment and redox buffering. Similar clustering of antioxidant traits along secondary PCA axes has been reported in recent multivariate analyses, where PC2 often reflects the magnitude of biochemical stress responses rather than direct productivity gains^48,49^.

The distinct positioning of the rhizobacterial consortium in the positive quadrants of both PC1 and PC2 indicates that this treatment elicited a balanced and integrative response under drought stress. Its close alignment with yield-related traits, chlorophyll content, relative water content, and antioxidant enzymes suggests that the consortium simultaneously enhanced physiological protection, biochemical defense, and growth processes. Such integrated responses are increasingly recognized as a hallmark of effective microbial consortia, which can modulate multiple plant pathways through complementary mechanisms, including improved nutrient acquisition, hormonal regulation, and stress signaling^54,55^.

In contrast, single inoculants exhibited more moderate and variable responses. The relatively better position of the BS treatment along PC1 suggests a greater influence on growth- and yield-associated traits. In contrast, PP and AC clustered closer to the origin, indicating partial alleviation of drought stress but limited integration across multiple trait domains. This pattern aligns with recent evidence showing that individual plant growth-promoting rhizobacteria often target specific physiological processes, while consortia provide broader functional coverage through synergistic interactions^56–58^.

The spatial separation of the control treatment along the positive side of PC1, but away from antioxidant-associated vectors, reflects optimal growth under non-stress conditions, with comparatively lower activation of stress defense pathways. Conversely, the drought treatment clustered in the negative region of both PC1 and PC2, closely associated with oxidative damage indicators, confirming the severe physiological disruption caused by water deficit in the absence of microbial intervention. Such clear segregation between stressed and unstressed treatments is commonly reported in PCA studies of drought responses, thereby validating the robustness of the multivariate approach in capturing treatment effects^45,52^.

Overall, the PCA results demonstrate that isolated improvements in single traits, but by coordinated regulation of water status, photosynthetic capacity, antioxidant defense, and growth processes, did not drive drought tolerance in rhizobacteria-treated plants. The superior positioning of the rhizobacterial consortium highlights its ability to integrate stress mitigation and productivity-related functions, thereby sustaining yield under drought stress more effectively than individual inoculants. These findings reinforce the idea that multi-strain microbial consortia offer a more resilient and comprehensive strategy to enhance crop performance in water-limited environments.

## Conclusion

The rhizobacterial consortium composed of *Azotobacter chroococcum*, *Pseudomonas putida*, and *Bacillus subtilis* consistently improved plant water status, membrane integrity, photosynthetic pigment retention, and antioxidant defense systems, while concurrently reducing oxidative damage under drought stress. These coordinated physiological and biochemical adjustments translated into enhanced vegetative growth, reproductive performance, and yield stability. Multivariate analyses, including correlation and principal component analyses, revealed that an interconnected physiological, biochemical, and morphological continuum governs drought tolerance in tomato. Traits related to plant hydration, chlorophyll preservation, and biomass accumulation were strongly associated with yield formation, whereas indicators of membrane disruption and oxidative stress exhibited clear antagonistic relationships. The prominent positioning of the consortium across both productivity- and defense-related trait axes highlights its superior capacity to harmonize stress mitigation with growth and yield maintenance compared with single-strain inoculations.

Overall, these findings demonstrate that multi-strain rhizobacterial consortia represent an effective and sustainable strategy for enhancing drought resilience in tomato production. By synchronizing water relations, antioxidant protection, and photosynthetic performance, such consortia offer a promising microbial-based approach for stabilizing crop productivity under increasingly water-limited conditions. Future research should focus on validating the long-term effectiveness of these consortia under field conditions, exploring their interactions with native soil microbiomes, and assessing their scalability and consistency across diverse agroecological zones within climate-resilient horticultural systems.

## Data Availability

The datasets generated and/or analyzed during the current study are available from the corresponding author on reasonable request.
